# Pain tolerance predicts human social network size

**DOI:** 10.1038/srep25267

**Published:** 2016-04-28

**Authors:** Katerina V.-A. Johnson, Robin I. M. Dunbar

**Affiliations:** 1 University of Oxford, Department of Experimental Psychology, South Parks Road, Oxford, OX1 3UD, UK

## Abstract

Personal social network size exhibits considerable variation in the human population and is associated with both physical and mental health status. Much of this inter-individual variation in human sociality remains unexplained from a biological perspective. According to the brain opioid theory of social attachment, binding of the neuropeptide β-endorphin to μ-opioid receptors in the central nervous system (CNS) is a key neurochemical mechanism involved in social bonding, particularly amongst primates. We hypothesise that a positive association exists between activity of the μ-opioid system and the number of social relationships that an individual maintains. Given the powerful analgesic properties of β-endorphin, we tested this hypothesis using pain tolerance as an assay for activation of the endogenous μ-opioid system. We show that a simple measure of pain tolerance correlates with social network size in humans. Our results are in line with previous studies suggesting that μ-opioid receptor signalling has been elaborated beyond its basic function of pain modulation to play an important role in managing our social encounters. The neuroplasticity of the μ-opioid system is of future research interest, especially with respect to psychiatric disorders associated with symptoms of social withdrawal and anhedonia, both of which are strongly modulated by endogenous opioids.

The origin of societies is considered one of the major evolutionary transitions^1^. This has been accomplished by numerous species but arguably no society is as widespread, complex and technologically advanced as our own. The human brain has evolved to thrive in social environments, providing us with the cognitive processing power to deal with our dynamic and intricate personal relationships^2^. However, there is limited understanding of the neurobiological processes underpinning human sociality. A growing number of studies highlight the important role played by endogenous opioid peptides, most notably β-endorphin, in affiliation and bonding in social animals such as rodents and primates, including humans^3^,^4^. This neuropeptide is released from the CNS and has the highest binding affinity for μ-opioid receptors, which are widely distributed in the brain^5^. Upon binding, β-endorphin induces analgesia and a sense of well-being^6^,^7^. The brain opioid theory of social attachment^8^ postulates that the endogenous μ-opioid system is fundamental to the establishment and maintenance of social bonds. Indeed, μ-opioid neurotransmission has been shown to modulate social motivation^4^ and plays a key role in attributing positive value to social interactions^9^. Specifically, the close relationship between the opioid and dopamine systems is integral to the rewarding nature of social interactions^10^.

Until relatively recently, experimental evidence supporting the role of the endogenous opioid system in modulating social behaviour mainly derived from the administration of opioids and opioid blockers^3^,^11^. For instance, humans given the μ-opioid antagonist naltrexone experience feelings of reduced social connection^12^. With advances in genetics, knockout technology has revealed that mice lacking the μ-opioid receptor gene show severe deficits in numerous facets of social behaviour, including interactions with conspecifics, communication and infant attachment^13^,^14^. Furthermore, there is increasing interest in the use of positron emission tomography (PET) scanning to measure activity of the μ-opioid system in relation to differences in social behaviour, both within individuals (Manninen *et al*. in prep) and between individuals^15^.

Since β-endorphin is a potent analgesic, indeed more so than the pain-relieving opiate drug morphine^16^, the primary hypothesis tested here was whether pain tolerance (as a proxy for activation of the μ-opioid system) predicts social network size. We tested this hypothesis in a population of healthy young adults (*n* = 101). The study involved a questionnaire relating to the two innermost social network layers (approximately corresponding to those individuals contacted at least once a week and once a month respectively), as well as collecting information on personality, sociodemographics and lifestyle. Since the blood-brain barrier is impermeable to β-endorphin, CNS endorphin levels can only be accurately determined by sampling cerebrospinal fluid^3^ via lumbar puncture, whilst measuring the μ-opioid system directly requires the use of PET scanning^17^. Instead, pain tolerance was assessed by means of a non-invasive, physical pain test (see Methods).

## Results

**Figure 1:**
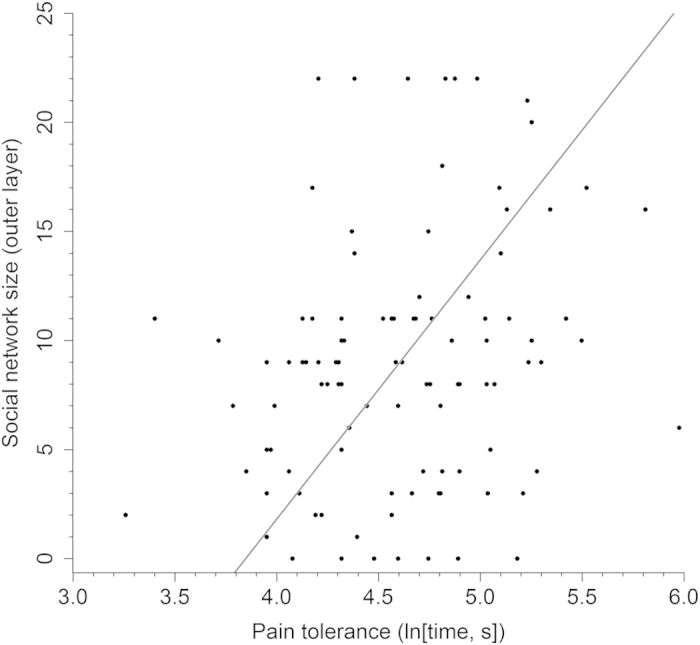
Relationship between pain tolerance and social network size. Pain tolerance is a significant predictor of an individual’s social network size (*P* = 0.010), especially the size of their outer network layer (*P* = 0.002) as depicted here. This represents those individuals contacted at least monthly, but less frequently than once a week. Pain tolerance is plotted as the natural log transformation of pain test time and the reduced major axis regression line is shown.

Multiple regression analysis (see Supplementary Tables S1–3) revealed pain tolerance to be a significant predictor of social network size (*P* = 0.010), in particular the size of an individual’s outer network layer (*P* = 0.002, Fig. 1). This corresponds to members of their network whom they are typically in contact with at least monthly but less frequently than once a week. The personality trait agreeableness also positively predicted network size but was negatively related to pain tolerance and thus proved not to mediate the above relationship (Supplementary Tables S4–6). Notably, there were no significant gender differences in pain test performance (*t*_99_ = −1.144, *P* = 0.255). Since pain tolerance is inferred from the length of time participants can endure the physical pain test, individuals with higher self-rated fitness performed significantly better, as anticipated (Supplementary Tables S4–6). However, fitter individuals also had smaller social networks, particularly the outer network layer (*P* = 0.021), and so fitness was not a confounding variable in the relationship between pain tolerance and network size. The analysis also revealed stress to be a significant negative predictor of outer social network size (*P* = 0.023), with individuals who reported higher stress levels having smaller networks.

## Discussion

Our results show that pain tolerance positively predicts social network size. This therefore supports our hypothesis that variation in the μ-opioid system underlies individual differences in sociality. These results are consistent with a recent PET imaging experiment demonstrating a correlation between μ-opioid receptor availability and attachment style, such that individuals showing greater avoidance of social attachment exhibit lower receptor densities^15^. Our findings are also in agreement with previous pain tolerance studies indirectly implicating the endogenous opioid system in human social bonding activities such as music-making^18^, dancing^19^ and laughter^20^. In addition, laughter has since been shown to correlate with elevated μ-opioid activity, as measured by PET scanning (Manninen *et al*. in prep). This suggests that tests of pain tolerance like that used in our study may indeed serve as a useful proxy for assessing activation of the μ-opioid system.

Variation in μ-opioid receptor signalling may be due to underlying differences in both endogenous opioid release and receptor density, though their relative contribution is yet to be fully determined. However, studies of oxytocin and vasopressin signalling in rodents have shown that CNS receptor densities strongly modulate the influence of these neuropeptides, irrespective of neuropeptide abundance^21^. In fact, analyses of post-mortem brain tissue and *in vivo* PET studies in humans have revealed a broad range of μ-opioid receptor densities within the population, differing by at least 30–50%^22^. Such variation is likely to considerably affect the potency of β-endorphin^11^. Genetic studies suggest these differences in receptor density are partly the result of a non-synonymous single-nucleotide polymorphism in the μ-opioid receptor gene (*OPRM1*), substituting aspartic acid for asparagine (A118G)^23^. This functional polymorphism is relatively common in the population, with the minor G allele having a frequency of 10–30%^24^, and is associated with reduced μ-opioid receptor expression^24^,^25^. The G allele has also been linked to increased social withdrawal^26^ and reactivity to social rejection^27^, as well as greater pain sensitivity and reduced relief from opiate drugs^28^. This is therefore in line with our proposition that variation in the μ-opioid system contributes to individual differences in both social behaviour and pain tolerance.

We acknowledge that use of pain tolerance as a proxy for μ-opioid receptor signalling, rather than its direct measurement, represents a limitation of our research. However, the μ-opioid system is critically involved in pain modulation^6^,^29^ and numerous PET studies implicate μ-opioidergic activation in both experimental and clinical pain settings^30^. Most notably, in humans undergoing a sustained muscular pain challenge, individuals exhibiting higher activity of the μ-opioid system report reduced sensory and affective pain^31^. A possible future direction that would benefit research in this field would be to combine PET scanning with a range of different pain tests to determine how reliably they can predict activity of the μ-opioid system. We also recognise the possible involvement of non-opioid signalling pathways, especially given the complex neurochemistry underlying pain responses^32^,^33^. In particular, oxytocin, vasopressin and endocannabinoids are all implicated in social behaviour^34^,^35^, as well as having analgesic effects^36^,^37^,^38^. Indeed, it is likely that they act in concert with β-endorphin^39^,^40^,^41^.

Further research is required to understand the causality of this relationship between pain tolerance and network size. It may be that individuals with genetic variants conferring enhanced μ-opioid neurotransmission derive greater reward from social interactions, thereby seeking more company. An alternative, though not mutually exclusive, explanation is that individuals leading lives rich in social interactions may release higher levels of endogenous opioids and/or have elevated receptor expression. However, we currently lack knowledge regarding the neuroplasticity exhibited by the μ-opioid system. This is of particular interest in relation to psychiatric disorders. Indeed, healthy females asked to sustain a sad mood for only 30 minutes show a reduction in μ-opioid receptor activation^42^. Thus prolonged sadness, as experienced by those suffering from depression, may over time lead to a significant fall in opioidergic signalling. We hypothesise that reduced μ-opioid activity may characterise the onset of conditions such as depression and schizophrenia, resulting in the common symptoms of anhedonia and social withdrawal. Indeed, endogenous opioids mediate hedonic experiences and are integral to our feelings of social connection^8^,^12^. In support of this, there is evidence of compromised μ-opioid receptor signalling in patients suffering from depression and schizophrenia^43^,^44^ and studies using rodent models of depression also implicate the μ-opioid system^45^.

With respect to the other notable results of our analysis, fitness was primarily included in the regression model to account for its influence on pain tolerance but revealed an interesting and novel negative relationship with network size. This indicates a trade-off between leading a socially active versus a physically active life. Beyond the obvious constraint of time, this relationship may reflect our underlying neurobiology such that individuals who exercise more may have greater reliance on this method of promoting β-endorphin release, rather than through social interactions. Though exercise is frequently prescribed as a treatment for depression, perhaps focus should also be placed on strengthening and expanding an individual’s social ties.

The relationship reported here between stress and network size may reflect the beneficial effects of social support in dealing with stressful situations^46^, since measures of social support often correlate with social network size^47^. Interestingly, one study found that the number of Facebook friends (a known correlate of real-world social network size^48^) is associated with enhanced perceptions of social support and reduced stress^49^. Whether online social networks play a role in relieving stress (or even intensifying it) over and above an individual’s actual social interactions remains uncertain. However, an alternative interpretation of our data is that stressed individuals find less time for social engagement and thus their network decreases in size.

Understanding the biological causes of variation in social network size is of particular interest given the robust association between an individual’s social support and their health, ranging from functioning of their immune, endocrine and cardiovascular systems^46^ to myelin integrity^50^. Interestingly, it is an individual’s perceived level of social support that may often be a more reliable indicator of their health status^46^,^51^. Compared to other lifestyle factors, we have limited understanding of the mechanisms via which sociality influences morbidity and mortality risk^52^, though reduced activation of the neuroendocrine stress response likely plays a significant role in both humans^51^,^53^ and animals^54^. Since β-endorphin is known to alleviate the stress response^55^ and protect against inflammation and cancer^56^, the activity of an individual’s endogenous μ-opioid system may have important consequences for their health. However, such a direct interaction between social and somatic health is yet to be explored.

In summary, there is substantial evidence that μ-opioid neurotransmission influences sensitivity not only to our physical environment but also our social one. This study adds to previous research implicating the μ-opioid system as a key neural substrate upon which human sociality has evolved. A better understanding of the neurobiological mechanisms underpinning our social lives is imperative, especially since our technology-driven world is rapidly changing the nature of social relationships and certainly outpacing any biological adaptations. Sociality is clearly of adaptive value to our species, yet in this digital era deficiencies in our social interactions may be one of the overlooked factors contributing to the declining health of our modern society.

## Methods

### Participants

The study was advertised for healthy adults aged 18–35 years, recruited predominantly from the University of Oxford. Exclusion criteria were recreational drug use or drug replacement therapy. Participants were requested not to consume alcohol (within 24 hours) or smoke (within 3 hours) prior to the study, given the analgesic properties of these substances.

The mean age of respondents was 21.7 years (range = 18–34 years). In total 107 subjects (30 males and 77 females) took part in the study. Six data points were excluded from the analysis due to either questionnaire inadequacies or failure to perform the pain test correctly. The study was approved by the University of Oxford’s Medical Sciences Inter-Divisional Research Ethics Committee and the methods were carried out in accordance with the approved guidelines. All participants gave written informed consent.

### Questionnaires

The social network questionnaire was designed to collect data relating to the two innermost layers of a participant’s social network, corresponding to those individuals contacted on a weekly and monthly basis respectively^57^. The 50-item IPIP (International Personality Item Pool) inventory^58^ was used to score individuals on each of the ‘Big-Five’ personality traits (openness, conscientiousness, extraversion, agreeableness and neuroticism). Respondents also provided basic sociodemographic and health information, along with self-rated assessments of their fitness and stress levels.

### Pain tolerance test

Given the invasive nature of PET imaging, pain tolerance is often used as a conventional assay in studies of the endogenous opioid system^18^,^19^,^20^. Participants performed an isometric quadriceps exercise (commonly known as the wall sit test) which involves squatting against the wall with knees at a 90° angle and a straight back. They were asked to hold this position and endure the discomfort for as long as possible and the time was recorded to the nearest second. The main advantages of this pain test are that it is non-invasive, does not require any specialist equipment and is quick to conduct, with an average time of 113 s (range = 26–394 s).

### Statistical analyses

Analyses were performed using R 3.2.3 software^59^ and all tests were conducted with an α level of 0.05. The construction of general linear models was guided by the Akaike Information Criterion, incorporating pain tolerance, self-rated fitness, stress and agreeableness as predictors of network size. Where necessary the appropriate variables were transformed, including natural log transformation of the pain test times, such that model residuals were normally distributed (Shapiro-Wilk test, *P* > 0.05) and satisfied the assumption of homoscedasticity (non-constant variance test, *P* > 0.05). The relationship between pain tolerance and social network size was plotted using the reduced major axis regression line which minimises the sum of the product of residuals in both the x and y directions. Partial correlations between variables were also calculated and the absence of multicollinearity confirmed using variance inflation factors. For analysis involving comparison of means, Student’s two-sample t-test (two-tailed) was conducted.

## Additional Information

**How to cite this article**: Johnson, K. V.-A. and Dunbar, R. I. M. Pain tolerance predicts human social network size. *Sci. Rep*. **6**, 25267; doi: 10.1038/srep25267 (2016).

## Supplementary Material

Supplementary Information
