# Supplementary material for: Pain tolerance predicts human social network size
Source: Sci Rep. 2016 Apr 28;6:25267. doi: 10.1038/srep25267 (PMC4848525; doi:10.1038/srep25267)
Supplement: Supplementary Information [file srep25267-s1.pdf]

Supplementary Information

Pain tolerance predicts human social network size

Katerina V.-A. Johnson[1,*] and Robin I. M. Dunbar[1]

[1]University of Oxford, Department of Experimental Psychology, South Parks Road, Oxford, OX1 3UD

*katerina.johnson@pmb.ox.ac.uk

Supplementary Table S1. Regression statistics for predictors of social network size.

Predictor	Estimate ± SE	*t* value	*P* value	ΔR^2
Pain tolerance	0.255 ± 0.096	2.645	0.010*	0.066
Fitness	-0.048 ± 0.025	-1.940	0.055	0.035
Stress	-0.027 ± 0.022	-1.226	0.223	0.014
Agreeableness	0.016 ± 0.008	2.013	0.047*	0.038
			Total R^2	= 0.101

Supplementary Table S2. Regression statistics for predictors of inner social network size.

Predictor	Estimate ± SE	*t* value	*P* value	ΔR^2
Pain tolerance	0.091 ± 0.084	1.080	0.283	0.012
Fitness	-0.008 ± 0.022	-0.381	0.704	0.002
Stress	-0.003 ± 0.019	-0.146	0.884	<0.001
Agreeableness	0.009 ± 0.007	1.318	0.191	0.018
			Total R^2	= 0.026

Supplementary Table S3. Regression statistics for predictors of outer social network size.

Predictor	Estimate ± SE	*t* value	*P* value	ΔR^2
Pain tolerance	3.844 ± 1.189	3.232	0.002*	0.093
Fitness	-0.721 ± 0.307	-2.349	0.021*	0.049
Stress	-0.625 ± 0.270	-2.318	0.023*	0.048
Agreeableness	0.210 ± 0.096	2.194	0.031*	0.043
			Total R^2	= 0.150

*Denotes statistical significance at $\alpha = 0.05$

Supplementary Table S4. Partial correlations between variables in S1 regression model.

	Total network size	Pain tolerance	Fitness	Stress
Pain tolerance	0.278*	-	-	-
Fitness	-0.185	0.350*	-	-
Stress	-0.185	0.164	-0.287*	-
Agreeableness	0.202*	-0.216*	0.169	0.208*

Supplementary Table S5. Partial correlations between variables in S2 regression model.

	Inner network size	Pain tolerance	Fitness	Stress
Pain tolerance	0.115	-	-	-
Fitness	-0.038	0.318*	-	-
Stress	-0.041	0.124	-0.263*	-
Agreeableness	0.098	-0.179	0.140	0.181

Supplementary Table S6. Partial correlations between variables in S3 regression model.

	Outer network size	Pain tolerance	Fitness	Stress
Pain tolerance	0.313*	-	-	-
Fitness	-0.233*	0.365*	-	-
Stress	-0.230*	0.183	-0.302*	-
Agreeableness	0.219*	-0.226*	0.181	0.219*

Supplementary Table S7. Variance inflation factors (VIF) confirming absence of multicollinearity between predictors.

Predictor	Pain tolerance	Fitness	Stress	Agreeableness
VIF	1.132	1.181	1.098	1.060

*Denotes statistical significance at $\alpha = 0.05$
